# Early postoperative prediction of the risk of distant metastases in medullary thyroid cancer

**DOI:** 10.3389/fendo.2023.1209978

**Published:** 2023-11-21

**Authors:** Yuhan Zhang, Qing Zhou, Guang Chen, Shuai Xue

**Affiliations:** General Surgery Center, Department of Thyroid Surgery, The 1^st^ Hospital of Jilin University, Changchun, China

**Keywords:** medullary thyroid carcinoma, high risk, distant metastases, predictive model, nomogram

## Abstract

**Purpose:**

The purpose of this study was to develop and validate a nomogram for estimating the risk of distant metastases (DM) in the early postoperative phase of medullary thyroid cancer (MTC).

**Patients and methods:**

We retrospectively reviewed cases of patients diagnosed with MTC from the Surveillance, Epidemiology, and End Results (SEER) database from 2007 to 2017. In addition, we gathered data on patients who diagnosed as MTC at Department of Thyroid Surgery in the First Hospital of Jilin University between 2009 and 2021. Four machine learning algorithms were used for modeling, including random forest classifier (RFC), gradient boosting decision tree (GBDT), logistic regression (LR), and support vector machine (SVM). The optimal model was selected based on accuracy, recall, specificity, receiver operating characteristic curve (ROC), and area under curve (AUC). After that, the Hosmer-Lemeshow goodness-of-fit test, the brier score (BS) and calibration curve were used for validation of the best model, which allowed us to measure the discrepancy between the projected value and the actual value.

**Results:**

Through feature selection, we finally clarified that the following four features are associated with distant metastases of MTC, which are age, surgery, primary tumor (T) and nodes (N). The AUC values of the four models in the internal test set were as follows: random forest: 0.8786 (95% CI, 0.8070-0.9503), GBDT: 0.8402 (95% CI, 0.7606-0.9199), logistic regression: 0.8670(95%CI,0.7927-0.9413), and SVM: 0.8673 (95% CI, 0.7931-0.9415). As can be shown, there was no statistically significant difference in their AUC values. The highest AUC value of the four models were chosen as the best model since. The model was evaluated on the internal test set, and the accuracy was 0.84, recall was 0.76, and specificity was 0.87. The ROC curve was drawn, and the AUC was 0.8786 (95% CI, 0.8070-0.9503), which was higher than the other three models. The model was visualized using the nomogram and its net benefit was shown in both the Decision Curve Analysis (DCA) and Clinical Impact Curve (CIC).

**Conclusion:**

Proposed model had good discrimination ability and could preliminarily screen high-risk patients for DM in the early postoperative period.

## Introduction

As of 2020, there will be 586,000 new cases of thyroid cancer worldwide, making up 3% of all cancer cases. Thyroid cancer incidence has consistently climbed over the previous 30 years (1). Medullary thyroid cancer (MTC) is a neuroendocrine tumor that develops from C cells in the thyroid gland and its incidence rate is low, accounting for just 2-4% of all thyroid cancers (2). The main treatment of MTC is surgery (3). As MTC is more aggressive and prone to recurrence and metastases compared to differentiated thyroid cancer (DTC) (4), regular postoperative follow-up is crucial for diagnosed patients. But due to differences in patient condition, tumor characteristics, and treatment strategies, individualized follow-up plans need to be tailored. When MTC patients are being followed up after surgery, some distant metastases may not be identified in a timely manner due to various reasons, resulting in poor prognosis. For example, although some typical patients may present with flushing and diarrhea when they have distant metastases (5), we have found in clinical practice that these symptoms may do not receive sufficient attention from postoperative patients because of the lack of specificity. Additionally, many atypical patients may have no specific symptoms. Moreover, calcitonin (Ctn) and carcinoembryonic antigen (CEA) are effective biomarkers to monitor tumor recurrence and distant metastases. But there are still some disadvantages about Ctn and CEA. The lengthy half-life of serum calcitonin may render early Ctn detection inefficient in accurately evaluating surgical efficacy, particularly when some patients have poor liver or kidney function or have high preoperative Ctn levels (6). Ctn doubling time requires a certain amount of time to elapse. For some non-secretory MTC, with double negative markers at the time of diagnosis and at the relapse, Ctn and CEA are not reliable biomarkers anymore (7, 8). The specificity and sensitivity of CEA are both inferior to Ctn, so we cannot just use CEA to determine whether a tumor has distant metastasis.

Given the aforementioned, we need a way to estimate individualized patient’s risk of distant metastases in the early postoperative period (within 3 months after surgery). If we can make a preliminary judgment of the risk of distant metastases based on the patient’s basic information, surgical strategy, and postoperative pathological report, patients will receive more accurate and personalized postoperative recommendations. Clinicians should alter their follow-up strategies for patients who have a high risk of distant metastases by decreasing the follow-up intervals and doing routine imaging tests. Follow-up intervals can be appropriately extended for individuals who have a low risk of metastases. Although relevant research on this issue has been carried out (9), we have found several shortcomings: firstly, they did not consider the impact of surgical strategy selection on distant metastases in MTC; secondly, there are many missing values in the SEER database, and they did not provide a detailed explanation of how to deal with missing values in their study; thirdly, their research lacks external validation. Therefore, based on previous research findings, we aim to further improve the research design and better address the relevant issues.

With the proposal of the concept of precision medicine (10), the idea of clinical doctors performing the same follow-up management on all patients has become outdated. There may be a possibility of over-diagnostics of low-risk patients and untimely treatment of high-risk patients. Therefore, we designed a retrospective study based on machine learning algorithms to evaluate the risk of distant metastases in the postoperative period in MTC patients.

## Materials and methods

### Data collection

The data for this study were collected from two sources: the Surveillance, Epidemiology, and End Results (SEER) database (n=2519) and the First Hospital of Jilin University (n=133).

The retrospective selection was carried out on case data from SEER between 2007 and 2017 based on International Classification of Diseases (ICD) code C73.9 and ICD-O-3 Hist/behave, malignant encoding of 8510, 8347, 8346, and 8345 for patients with MTC. Features related to distant metastases were preliminarily selected, including population features such as gender, age at diagnosis, and race, as well as tumor features such as “Primary Site - labeled, ICD-O-3 Hist/behav, malignant, RX Summ–Surg Prim Site (1998+), RX Summ–Scope Reg LN Sur (2003+), RX Summ–Surg Oth Reg/Dis (2003+), Reason no cancer-directed surgery, CS lymph nodes (2004-2015), Regional nodes examined (1988+), Regional nodes positive (1988+), SEER Combined Mets at DX-bone (2010+), SEER Combined Mets at DX-brain (2010+), SEER Combined Mets at DX-liver (2010+), SEER Combined Mets at DX-lung (2010+), CS tumor size (2004-2015), CS extension (2004-2015), and CS site-specific factor 1 (2004-2017 varying by schema)”.

Patient data from the electronic medical records database at our hospital were screened for pathology reports from 2009 to 2021 that indicated MTC. Through telephone follow-up survey so that the patients’ survival status and distant metastases were discovered.

Patients were not included in the analysis if their distant metastases status was unknown or missing. Ultimately, the SEER dataset included 1901, while the dataset from our hospital included 111. The data from SEER were divided into three sets: a training set (n=1332, 70%), an internal testing set (n=569, 30%), and the data from our department were split out as an external validation set (n=111).

Definition: Extent of surgery: no surgery (code 0): patients was diagnosed with MTC by fine-needle aspiration (FNA), and persisted in refusing surgical treatment despite being fully informed of the disease’s harm by the doctor. Total thyroidectomy (code 2): removal left and right lobes and isthmus of the thyroid gland. Non-total thyroidectomy (code 1): local tumor destruction, removal of less than a lobe including local surgical excision and removal of a partial lobe only, lobectomy and/or isthmectomy including lobectomy only and isthmectomy only and lobectomy with isthmus, removal of a lobe and partial removal of the contralateral lobe, subtotal or near total thyroidectomy.

Extent of lymph node dissection: N0: there are no evidence for metastases of reginal lymph nodes. N1a: anterior compartment group including level VI and level VII. N1b: cervical lymph nodes except for central compartment.

### Data preprocessing

All textual information was converted to numerical codes. For SEER data, we reclassified “ICD-O-3 Hist/behav, malignant” as follows: 8510/3: Medullary carcinoma, NOS, and 8345/3: Medullary carcinoma with amyloid stroma were coded as 0 for not merging with other types of tumors, while all other types of tumors were merged and coded as T staging was determined based on “CS tumor size (2004-2015)” and “CS extension (2004-2015)”, with T1, T2, T3, and T4 stages. N staging was determined based on “CS lymph nodes (2004-2015)” “regional nodes examined (1988+)” and “regional nodes positive (1988+)”, with N0, N1a, N1b, N1 stages. Metastases was determined based on “SEER Combined Mets at DX-bone (2010+)”, “SEER Combined Mets at DX-brain (2010+)”, “SEER Combined Mets at DX-liver (2010+)”, “SEER Combined Mets at DX-lung (2010+)”, and “CS mets at dx (2004-2015)”, and coded as either metastases or non-metastases, with 1 and 0 respectively. Surgery was classified as none surgery, non-total thyroidectomy, or total thyroidectomy and is represented by 0, 1, and 2, respectively. Lymph nodes dissection was classified as dissection (code 1) and no dissection (code 2). To address missing data in our study, we utilized a multivariate imputation strategy implemented in Python (version 3.9) to impute missing values in both datasets obtained from the SEER database and our hospital. This method involved estimating missing values based on patterns in the available data, resulting in two complete datasets that could be used for further analysis. By utilizing this approach, we were able to maximize the amount of available data. We used the SEER dataset for model training and testing, and the hospital dataset for external model validation. The training and testing sets of the SEER dataset were split in a 7:3 ratio. Due to the scarcity of positive events in the training set, which is not conducive to algorithm learning, we balanced the training set data to roughly equalize the proportion of 0 and 1 in the target.

### Statistical methods

In this study, we compared the clinical baseline characteristics of the training set and the internal test set by using SPSS, and completed the model training and validation by using Python.

Irrelevant features such as patient ID and the year of diagnosis, and features with low variances, such as primary we removed. We then used a tree-based feature selection method to determine the importance of each remaining feature and selected the most important ones, including age, surgery, T staging, N staging, and metastases.

Four models were trained using the random forest classifier (RFC), gradient boosting decision tree (GBDT), logistic regression (LR), and support vector machine (SVM). Grid search was employed to select the optimal parameters for each model. Accuracy, recall, specificity, and area under curve (AUC) with 95% confidence intervals (CI) were calculated for each model on the testing set, and the receiver operating characteristic curve (ROC) was plotted. Based on a comprehensive consideration of these indicators, the best-performing model was adopted. We used a nomogram to depict the model and related risk factors. The calibration of the prediction model was assessed using the Hosmer-Lemeshow goodness-of-fit test and calibration curve; A P value of 0.05 or higher suggests that the calibration is good. The brier score (BS) is used to assess the accuracy of the prediction model by measuring the mean squared difference between the anticipated probability and the true condition. Some studies suggest that a model with a BS greater than 0.25 may have poor predictive performance (11). The clinical efficacy of the model was then evaluated using threshold probability and net benefit following decision curve analysis (DCA) and clinical impact curve (CIC) on the model.

In both data sets, the normal distribution test and the Mann-Whitney test (U test) were used for quantitative data. The U-test was also used for ordered variables in the qualitative data. For the unordered variables, the chi-square test and Fisher’s exact test were used.

## Results

### Demographics and patient characteristics

A summary of patient characteristics and baseline comparisons can be found in **Table 1**.

**Table 1:** Comparison of clinical and pathological characteristics of the training and test sets.

	Training set	Internal testing set	P value
Metastasis (yes/no)	126/1206	38/531	0.05
Sex (male/female)	561/771	232/337	0.636
age	55±16	50±12	0.633
Race (white/black/asian)	1117/120/95	477/55/37	0.913
Other_tumors (yes/no)	90/1242	29/540	0.386
Surgery (none/non-total/total)	117/110/1105	43/50/476	0.868
LND (yes/no)	929/403	404/65	0.537
T (T1/T2/T3/T4)	676/313/264/79	311/130/94/34	0.600
N (N0/N1a/N1b)	811/201/320	363/68/138	0.832
Multi focus (yes/no)	437/895	176/393	0.382

LND, lymph node dissection.

### Prognosis review

In this study, we conducted regular follow-up of our hospital’s patients, with an average follow-up period of 40 months, range 2-144 months. During the follow-up, we identified three patients who developed distant metastases after surgery. In contrast, the SEER database had an average follow-up period of 60 months, range 0-143 months.

### Feature selection and model evaluation

**Figure 1:**
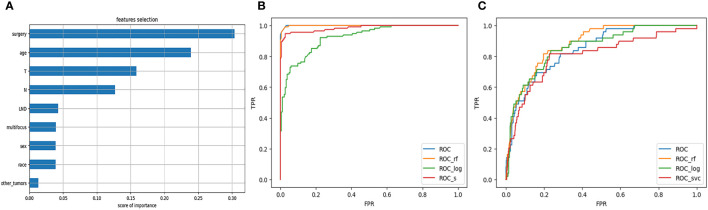
**(A)** Feature selection. **(B)** ROC curve of the model on the training set. **(C)** ROC curve of the model on the testing set.

Through the feature selection method described above, we obtained four highly correlated features: surgery, age, T stage, and N stage. The result of feature selection is shown in **Figure 1A**. For each model on the training and testing sets, model assessments are included in **Tables 2**, **3**. These tables include the AUC with 95% CI, accuracy, recall, and specificity. The ROC curves for the four models on the training and testing sets can be seen in **Figures 1B, C**. The AUC and 95% CI for the four models on the testing set were 0.8670 (95% CI, 0.7927-0.9413), 0.8402 (95% CI, 0.7606-0.9199), 0.8786 (95% CI, 0.8070-0.9503), and 0.8673 (95% CI, 0.7931-0.9415). We can see that these results indicate that all four models have good discriminative ability, with no statistically significant differences in predictive performance. Therefore, we selected the RFC model with the highest AUC as the final model.

**Table 2:** Evaluation of the model on the training set.

	AUC(95%CI)	Accuracy	Recall	Specificity
Random Forest	0.9961 (95%CI,0.9883-1.000)	0.97	0.94	0.98
GBDT	0.9994 (95%CI,0.9962-1.000)	0.98	0.98	0.98
Logistic Regression	0.9104 (95%CI,0.8738 -0.9470)	0.84	0.78	0.89
SVM	0.9055 (95%CI,0.8679-0.9430)	0.81	0.87	0.77

AUC, area under curve; GBDT, gradient boosting decision tree; SVM, support vector machine.

**Table 3:** Evaluation of the model on the testing set.

	AUC(95%CI)	Accuracy	Recall	Specificity
Random Forest	0.8786 (95%CI,0.8070-0.9503)	0.84	0.76	0.87
GBDT	0.8402 (95%CI,0.7606-0.9199)	0.82	0.74	0.87
Logistic Regression	0.8670 (95%CI,0.7927-0.9413)	0.87	0.61	0.89
SVC	0.8673 (95%CI,0.7931-0.9415)	0.81	0.71	0.81

AUC, area under curve; GBDT, gradient boosting decision tree; SVM, support vector machine.

### Nomogram development and validation

**Figure 2:**
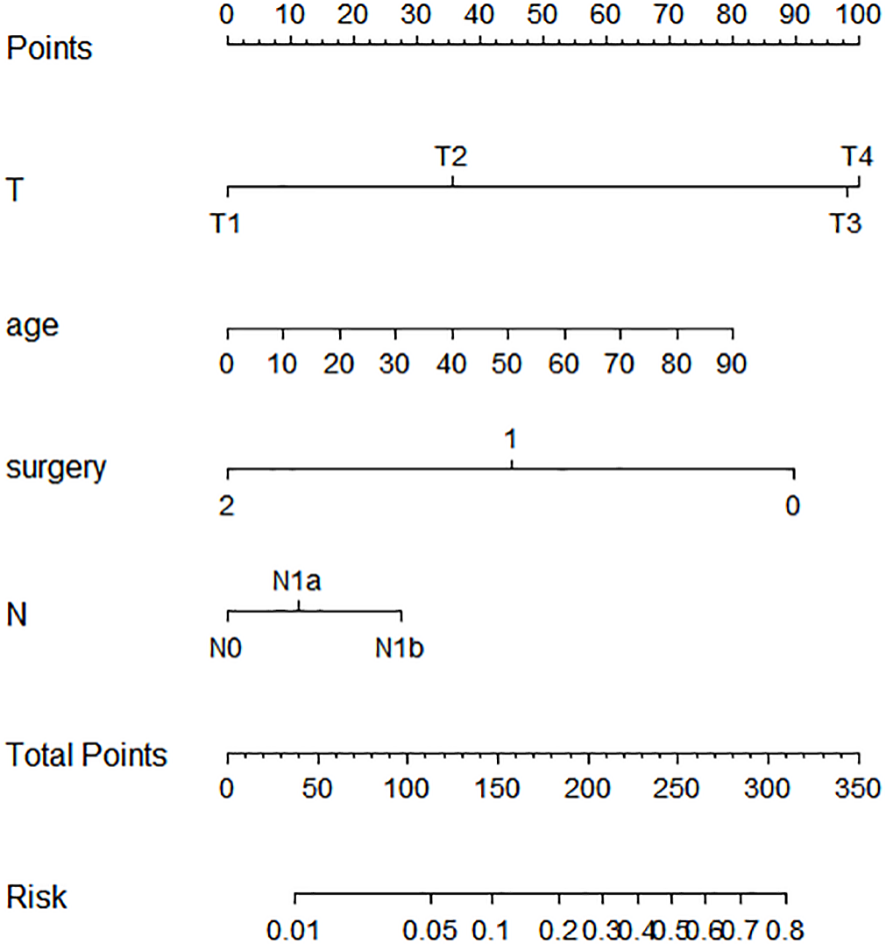
Nomogram for predicting the risk of distant metastases of medullary thyroid carcinoma. The nomogram is used by summing all points identified on the scale for each variable. The total points projected on the bottom scales indicate the probabilities of distant metastases in medullary thyroid carcinoma.

**Figure 3:**
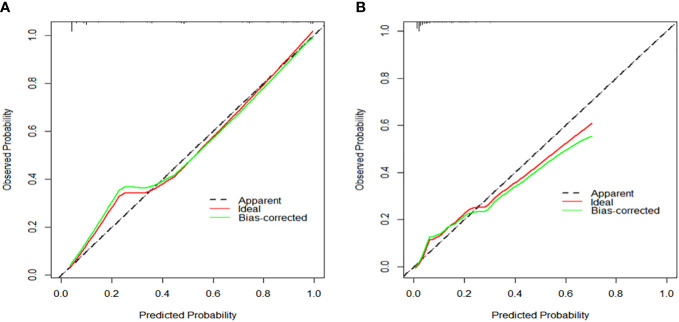
**(A)** Calibration curve to assess the calibration of the prediction model for the training set. **(B)** Calibration curve to assess the calibration of the prediction model for the testing set.

**Figure 4:**
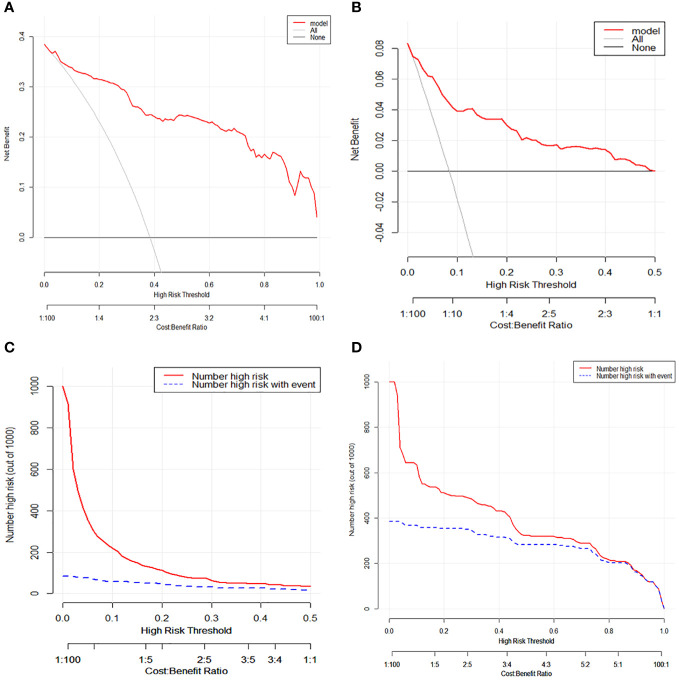
**(A)** Decision curve analysis and for distant metastases in medullary thyroid carcinoma patients on the training set. **(B)** Decision curve analysis for distant metastases in medullary thyroid carcinoma patients on the testing set. **(C)** Clinical impact curve for distant metastases in medullary thyroid carcinoma patients on the training set. **(D)** Clinical impact curve for distant metastases in medullary thyroid carcinoma patients on the testing set.

Based on the aforementioned findings, we determined that age, surgical approach, T stage, and N stage were risk factors for distant metastases for MTC. We then built a nomogram for estimating the probability of metastases based on these characteristics, which is shown in **Figure 2**. In this nomogram, we can see that older age, no surgery, small extent of surgery, higher T staging, higher N staging were related with distant metastases. From this figure, we can easily know that, the higher T staging or N staging the greater risk of distant metastases. No surgery or non-total thyroidectomy will also increase the risk of distant metastases. This figure also shows that the risk of distant metastasis shows an upward trend with the diagnosed age increasing. In the comprehensive assessment of transferred risks, T staging and surgical strategy have the greatest impact on the distant metastases, while N staging has the least impact on it. We used calibration curves to evaluate the early postoperative prediction of distant metastases risk in MTC. The results showed a good fit in both the training and internal testing sets which can be seen in **Figures 3A, B**. The P values for the training set and testing set in the Hosmer-Lemeshow goodness-of-fit test were both higher than 0.05, showing that the chosen model had been calibrated well. The BS score of the model was 0.102 in the training set and 0.059 in the internal testing set, both lower than 0.25, indicating good overall performance. As shown in **Figures 4A–D**, it can be seen that the net benefit of the model both the training and internal testing sets is higher than the extreme curve, and the range of selectable threshold values is relatively large, indicating good clinical value. Finally, we validated the final model on an external training set (clinical and pathological characteristics were shown in **Table 4**), with an AUC of 0.9105, accuracy of 0.84, recall of 1.0, and specificity of 0.83, demonstrating good predictive ability.

**Table 4:** Summary of clinical and pathological characteristics of the external validation set.

	External testing set
Metastasis (yes/no)	3/108
Sex (male/female)	37/74
age	50±11
Race (white/black/asian)	0/0/111
Other_tumors (yes/no)	72/39
Surgery (none/non-total/total)	1/31/79
LND (yes/no)	93/18
T (T1/T2/T3/T4)	54/43/12/2
N (N0/N1a/N1b)	39/31/41
Multi focus (yes/no)	19/92

## Discussion

MTC accounts for roughly 13.4% of all thyroid cancer fatalities and has a worse prognosis than DTC (12). According to prior research, the 10-year survival rate for MTC is approximately 96%, but the likelihood of distant metastases can be as high as 20%, and once it happens, the 10-year survival rate decreases dramatically to 40% (13). Parafollicular C cells are incapable of concentrating iodine, in contrast to DTC, hence radioactive iodine has no therapeutic benefit for MTC (14). It is crucial to identify DM early in order to take timely treatment. The most likely organ for MTC to metastasize is the lung (52.2%), followed by bone metastases (28.3%), mediastinal metastases (19.6%), and liver metastases (17.4%) (15). Imaging can be used to confirm metastases (16). Early imaging examinations such as ultrasound, chest CT, PET-CT, and bone scans can effectively detect metastatic lesions, which is beneficial for physicians to intervene early in patients with metastases and improve prognosis (17).

Currently, clinical guidelines recommend the concentration of Ctn and CEA in serum, as well as their doubling time, as independent risk factors for predicting disease prognosis (18). It is undeniable that the levels of Ctn and CEA and the multiplying time are highly related to disease recurrence and metastases. However, due to the characteristics of Ctn itself, there is a risk of not detecting metastases within 3 months after surgery. This research fills a void in the 3 months following surgery when there were no biochemical markers for predicting the likelihood of distant metastases in MTC patients. We included a data set of 111 MTC patients from our hospital from 2009 to 2021 as an external validation set. In the data set from the SEER, the metastases rate was approximately 8.66%, while in our hospital’s data set, the metastases rate was approximately 2.268%. We conducted follow-ups on patients in the external validation set and found that the model provided high scores for patients who were found to have distant metastases during follow-up. The validated results confirm the effectiveness of the model. In practical applications, patients with high scores should receive more attention from physicians after surgery, and when developing corresponding follow-up plans, the interval between follow-ups should be shortened, and more frequent imaging examinations should be considered.

Through our research, we have found that non-surgical treatment or incomplete thyroidectomy, older age, T3, T4, and lateral cervical lymph node metastases are all high-risk factors for distant metastases of MTC after surgery. A previous study on predicting MTC distant metastases did not mention the impact of the surgical approach on postoperative metastases (9). However, in our study, we found that the choice of surgical strategy and T staging are more relevant independent hazardous factor for MTC distant metastases. In a 29-year follow-up research, it was discovered that the degree of surgery was a significant risk factor that affected the prognosis of patients, and patients who underwent total thyroidectomy had better clinical results than those who underwent no surgery or simply unilateral thyroidectomy (13). All MTC patients are advised to have total thyroidectomy according to MTC treatment guidelines (19), which indirectly confirms our research findings. Previous literature has indicated that older age is one of the independent risk factors for distant metastases (9), and the probability of distant metastases increases significantly in patients over 55 years old (20), which indirectly indicates that older age increases the risk of distant metastasis of tumors. Our research showed that both the T and N stages of the tumor are positively correlated with the risk of distant metastases, meaning that the likelihood of distant metastases increases with stage. Other researchers have published articles mentioning that the T stage is a significant disadvantage factor for MTC distant metastases (21). A. Machens et al. discovered that individuals with lymph node metastases were more likely to acquire distant metastases, whether familial or sporadic, than those who did not in the research involving 1115 MTC patients (22). In addition, an article from the research of Kuo. Ej et al. mentioned that disease staging is an independent risk factor for fatal distant metastases in MTC (23), which indirectly confirms our conclusions. We analyse the results that the likelihood of developing distant metastases increases with tumor size and the extent of cervical lymph node metastases because the wider the scope of the tumor invasion, the more susceptible it is to invade the small blood vessels and within the thyroid, and the greater the probability of blood dissemination. In our study, although the metastatic spread of lymph nodes is a relevant factor for distant metastases of MTC, its importance was less significant compared to the other three variables. At the same time, we found that whether or not to perform neck lymph node dissection was only weakly correlated with MTC distant metastases. Currently, there is controversy over whether regular removal of lymph nodes in the neck should be performed. A number of doctors are positive about neck lymph node dissection, believing that it can bring positive effects. A reported article mentioned that all patients who were diagnosed with MTC without distant metastases should undergo central node neck (levels VI and VII) dissection (24). But a certain proportion of doctors believe that preventive lateral neck lymph node dissection cannot significantly improve patient prognosis without evidence of lateral neck lymph node metastases (25, 26). Because it is not difficult to see from the column chart that the impact of lymph node metastases on the risk of distant metastases is relatively small, we believe that routine lateral neck lymph node dissection is unnecessary in the absence of clinical evidence of lateral neck lymph node metastases. The remaining variables in our study did not show a strong correlation with MTC distant metastases.

In this study, we utilized the SEER database to train and test our model and validated it with our own data, achieving favorable results. Our calibration curve showed a good fit between predicted and observed outcomes, indicating that our model has good discriminatory ability (27). This conclusion was further confirmed through external validation.

Certainly, like the vast majority of studies, our study has some limitations. First, the SEER database contains a large number of missing values. Although we used multiple imputation to minimize the difference from the true situation, some errors are inevitable. Second, the SEER database lacks genetic information, and it is not possible to distinguish whether the included patients have a family history. So we cannot differentiate between sporadic and familial MTC for exploration. Third, the sample size of the external validation set is small, with only three cases of distant metastases. Fourth, the extent of LND is very important but the SEER database only contains information on the extent of lymph node involvement and does not subdivide the scope of lymph node dissection. As a result, we only included the degree of regional lymph node invasion in the model. In addition, the most selected hospitalized patients had a strong willingness for surgery, as a result, cases without surgery or undergoing unilateral thyroidectomy were similarly scarce. Therefore, the results obtained from external validation lack persuasiveness.

## Conclusion

Based on a large dataset of patients with MTC from SEER and the First Hospital of Jilin University, we have successfully developed a model for early postoperative prediction of distant metastases risk based on four independent risk factors, which is presented in the form of a nomogram. The predictive model performs well on the test set and can effectively screen high-risk patients within the Ctn half-life period after surgery, especially those with impaired liver or kidney function or with elevated preoperative Ctn levels leading to a prolonged Ctn half-life. Clinicians can use this model to assess the risk of distant metastases for each patient in a timely and effective manner after surgery, providing a reference for developing more accurate and personalized follow-up plans for each patient.

## Data availability statement

The raw data supporting the conclusions of this article will be made available by the authors, without undue reservation.

## Ethics statement

This study was approved by the Institutional Ethics Committee of First Hospital of Jilin University. Informed consents were given to these patients of our medical center in the study.

## Author contributions

All authors contributed to the article and approved the submitted version.
